# Lysosome‐Targeted Squaramide Anion Transporters

**DOI:** 10.1002/chem.71243

**Published:** 2026-06-09

**Authors:** Elba Feo, Edward York, Anthony Thai, William G. Ryder, Daniel A. McNaughton, Philip A. Gale

**Affiliations:** ^1^ School of Mathematical and Physical Sciences Faculty of Science University of Technology Sydney Sydney New South Wales Australia; ^2^ Faculty of Science and Engineering Zernike Institute For Advanced Materials University of Groningen Groningen The Netherlands; ^3^ School of Chemistry University of Sydney Sydney New South Wales Australia; ^4^ Monash Institute of Pharmaceutical Science Monash University Parkville Victoria Australia

**Keywords:** anion binding, anion transport, lysosome‐targeting, squaramides, supramolecular chemistry

## Abstract

Targeting anion transporters to specific organelles has previously been shown to be an effective strategy to enhance their cytotoxicities against cancerous cell lines. In this work a new family of squaramide anion transporters appended with two different lysosome targeting groups via a 1,4‐substituted benzene spacer were synthesised. Cell viability studies showed that the transporters containing the *N,N*‐dimethylamine targeting group exhibited the greatest cytotoxic effects despite possessing lower transport activities than the non‐targeted control compounds and those containing the morpholine targeting group. Subcellular localisation studies using anthracene‐labelled squaramides found that both targeting motifs successfully directed compounds to the lysosome, but the morpholine‐containing motif accumulated to a lesser extent.

## Graphical abstract

Lysosome‐targeted squaramide anion transporters elicit different biological effects depending on the targeting group employed.

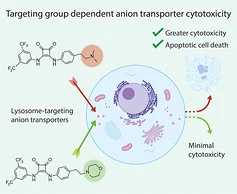

## 1 Introduction

Transmembrane anion transporters continue to garner interest due to their potential application as treatments for diseases such as cystic fibrosis [1, 2, 3] and cancer [4, 5, 6]. Current understanding of how anion transporters trigger cell death has been attributed to their ability to perturb intracellular ion concentrations. This causes osmotic stress, triggering the generation of reactive oxygen species and apoptosis via a caspase dependent pathway [4, 5]. Additionally, a number of chloride transporters have been shown to inhibit autophagy by decreasing lysosomal Cl^−^ concentrations via H^+^/Cl^−^ cotransport, causing an increase in lysosomal pH. As lysosomes require an acidic environment to break down cellular material, this deacidification impairs the autophagic recycling of cellular components, which contributes to the cytotoxicity of these compounds.

We have previously shown that targeting anion transporters to specific organelles can enhance cytotoxicity compared to non‐targeted analogues [7]. In a series of organelle targeting urea‐based anionophores, lysosome‐targeted compounds were found to exhibit the greatest enhancement in cytotoxicity towards A549 human lung cancer cells (IC_50_< 5 µM) compared to a non‐targeted analogue (IC_50_ = 12 µM), in a 24 h alamarBlue assay [7].

**FIGURE 1:**
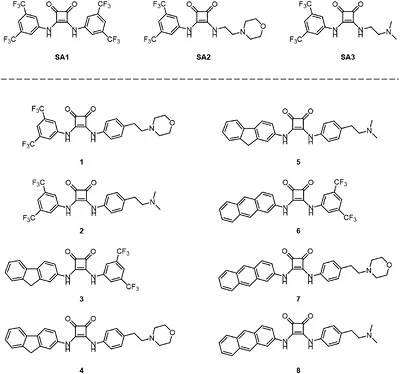
The structure of **SA1**, **SA2**, **SA3**, and squaramides **1**–**8**.

Squaramides are a potent anion transporter scaffold that can elicit significant cytotoxic effects [4, 5]. A study comparing the transport efficacy of squaramide and urea anionophores showed the 3,5‐bis‐CF_3_‐phenyl squaramide **SA1** (Figure 1) demonstrated a ∼30‐fold increase in Cl^−^ transport efficiency compared to its urea analogue [8]. Targeting squaramides to the lysosome using morpholine groups has previously been investigated by Chen and co‐workers [9, 10, 11]. Interestingly, they reported a series of morpholine‐appended squaramides (including **SA2** in Figure 1) that were able to efficiently deacidify the lysosomes of HeLa cells, yet exhibited no cytotoxicity [9]. This discrepancy could be attributed to the short alkyl linker to the morpholine group in **SA2**, allowing for intramolecular hydrogen bonding between the morpholine's tertiary amine to the squaramide NHs, which could interfere with anion binding and transport processes [12].

We propose that distancing the targeting group from the squaramide motif via a short rigid spacer would eliminate the possibility of unwanted intramolecular hydrogen bonding and increase the cytotoxicity of the squaramides. Furthermore, the addition of an aromatic spacer results in a greater electron withdrawing effect than the alkyl chain, which should increase the acidity of the squaramide NHs, and may increase the binding affinity of the squaramides to Cl^–^ [13]. Herein we present a series of lysosome‐targeted squaramides in which the targeting group is separated from the squaramide motif via a 1,4‐substituted benzene spacer. We discovered that separation of the targeting group from the central motif improved chloride binding compared to the corresponding spacer‐free control compounds (**SA2** and **SA3**, Figure 1), demonstrating the importance of considering potential intramolecular interactions in the design of anion transporters. *N,N*‐Dimethylamine‐appended transporters were also found to be more cytotoxic than the morpholine analogues, suggesting the targeting group can significantly impact the cellular effects of anion transporters. We believe these findings will help inform the design of future anion transporters to achieve specific biological outcomes.

## 2 Results and Discussion

### 2.1 Synthesis

**SCHEME 1:**
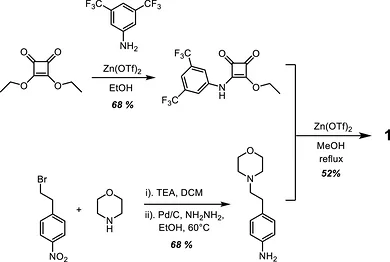
Synthesis of morpholine‐appended squaramide **1**.

The targeted‐squaramides were decorated with well‐established lysosome targeting groups, *N*,*N*‐dimethylamine and morpholine, which undergo reversible protonation at physiological pH driving their accumulation in the acidic lysosomal lumen. To visualise the intracellular localisation of the squaramides, fluorescent motifs were directly attached the central anion‐binding scaffold. Fluorene and anthracene moieties were selected as appropriate fluorophores to append to the squaramides due to their favourable lipophilicity and high compatibility with commercially available live‐cell organelle stains. The library of squaramides **1**–**8** were synthesised in 2–4 steps using adapted literature methods. Initially, diethyl squarate was reacted with various aromatic amines in the presence of a Zn(OTf)_2_ Lewis acid catalyst to give the monosubstituted squaramide intermediates [14]. The targeting group‐appended anilines were synthesised according to literature procedures starting from 4‐nitrophenethyl bromide (Scheme 1) [15]. The monosubstituted squaramides were then reacted with the targeted anilines to afford the final compounds (**1**–**8**). For the fluorene and anthracene analogues, a 19:1 toluene:DMF solvent mixture was required for the final step due to the lower solubility of their monosubstituted squaramide precursors. *N,N*‐Dimethyl amine targeted compounds **2**, **5**, and **8** were initially isolated as triflate‐containing compounds. An additional wash with triethylamine followed by water and methanol was required to isolate the compounds as the free base.

### 2.2 Anion Binding Studies

Squaramide‐based anion transporters facilitate transmembrane Cl^−^ transport via a mobile carrier mechanism, in which Cl^−^ is reversibly bound via convergent hydrogen bonds with the squaramide NHs to produce a membrane‐permeable complex [8]. Since anion binding is a key determinant of transport activity, the Cl^−^ binding affinities of squaramides **1**–**8**, **SA2**, and **SA3** were evaluated using ^1^H NMR titrations in DMSO‐*d_6_*/0.5% water. Solutions of the compounds were titrated with up to 200 equiv. of tetrabutylammonium chloride (TBACl). Large downfield shifts of the squaramide NH resonances and smaller shifts of some of the aromatic CH resonances were observed upon the addition of TBACl for all compounds.

The change in chemical shift of the NH and CH resonances upon Cl^−^ binding was fitted to various receptor:guest binding models using the Bindfit applet [16, 17]. Fitting to a 1:1 model was found to result in a good fit for all compounds. The highest binding affinity was found for compound **6** with a binding constant of 546 M^−1^, followed by **2** with a *K*_a_ of 468 M^−1^ (Table 1). Compounds containing the 3,5‐bis‐CF_3_ group (**1**, **2**, **3**, and **6**) exhibited higher binding affinities than those without, highlighting the importance of the increased acidity of the squaramide protons for anion binding, induced by the strong electron‐withdrawing nature of this substituent. This trend is consistent with potent transporter **SA1**, which possesses two 3,5‐bis‐CF_3_ groups and bound Cl^−^ strongly with a *K*_a_ of 643 M^−1^ [8].

**TABLE 1:** The stability constants (M^−1^) for the complexation of receptors **1**–**8** and Cl^−^ (as TBA salt) in DMSO‐*d_6_*/0.5% water, the EC_50_ values from the HPTS transport assay, and the IC_50_ values from the cell viability assay in MDA‐MB‐231 human breast cancer cells and BEAS‐2B human non‐cancerous bronchial epithelial cells.

Compound	*K*_a_ (M^−1^)^a^	EC_50_ (mol%)^b^	IC_50_ (µM) MDA‐MB‐231^c^	IC_50_ (µM) BEAS‐2B^d^
1	354	0.28	71.89	20.20
2	468	1.44	10.94	12.55
3	378	0.03	Non‐cytotoxic	Non‐cytotoxic
4	305	1.16	Non‐cytotoxic	Non‐cytotoxic
5	294	2.16	10.68	10.31
6	546	0.06	Non‐cytotoxic	Non‐cytotoxic
7	137	—^e^	Non‐cytotoxic	Non‐cytotoxic
8	150	— ^e^	21.80	11.17

^a^ All errors <8%.

^b^ EC_50_ at *t* = 200 s, shown as transporter:lipid molar percent (mol%).

^c^ Half maximum inhibitory concentration on MDA‐MB‐231 human breast cancer cells after incubation for 72 h.

^d^ Half maximum inhibitory concentration on BEAS‐2B human non‐cancerous bronchial epithelial cells after incubation for 72 h.

^e^ Transport activity did not reach 50% Cl^–^ efflux at the highest tested concentrations so full dose‐response curves could not be attained.

Notably, transporter **1** (354 M^−1^) and **2** (468 M^−1^) have higher binding affinities than **SA2** (187 M^−1^, Figure S44) and **SA3** (116 M^−1^, Figure S46), demonstrating that separating the targeting group from the binding motif via a rigid 1,4‐substituted spacer is an effective strategy for improving binding affinity of these squaramides.

### 2.3 Anion Transport Studies

**FIGURE 2:**
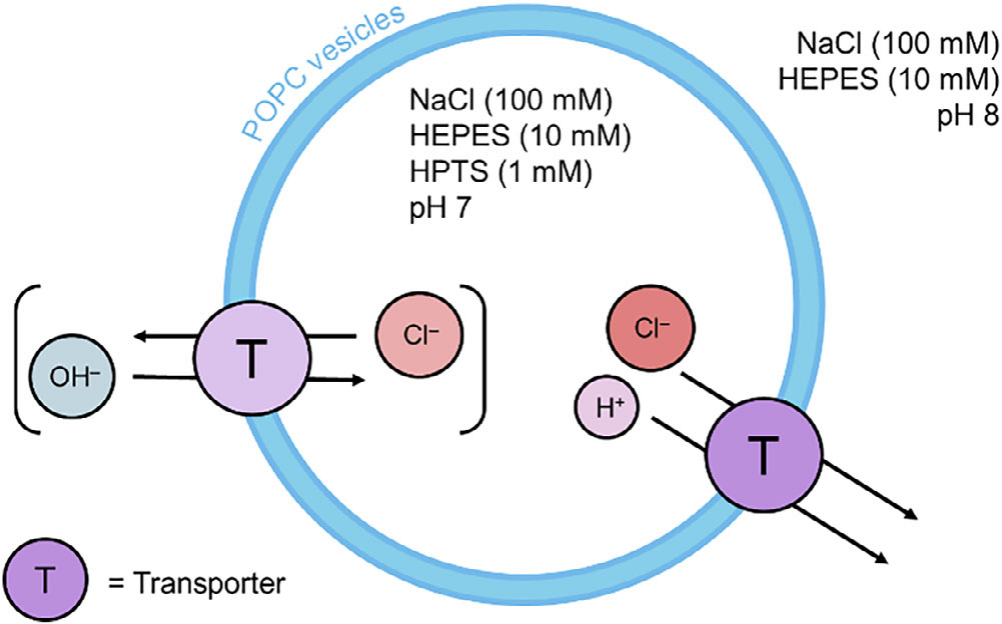
Schematic of the HPTS transport assay used to measure Cl^−^ transport by squaramides **1**–**8**. POPC vesicles containing HEPES (10 mM) buffered NaCl (100 mM) and HPTS (1 mM) were suspended in HEPES (10 mM) buffered NaCl (100 mM) at pH 7. To start the experiment a base pulse of NaOH (0.5 M) was added to increase the external pH to pH 8. Transport via H^+^/Cl^−^ symport or functionally equivalent OH^−^/Cl^−^ antiport mechanisms results in intravesicular pH changes, which can be monitored by comparing the fluorescence intensity of HPTS in its protonated and deprotonated forms (F_460_/F_403_).

**FIGURE 3:**
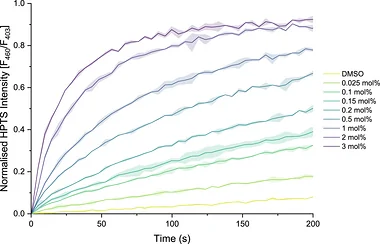
Dose response studies for compound **1** using the HPTS assay. Each data point is the average of two repeats with error bars to show the standard deviation. A run of pure DMSO was used as a control.

The anion transport activity **1**–**8** was then investigated using a fluorescent 8‐hydroxypyrene‐1,3,6‐trisulfonic acid (HPTS) transport assay [18]. Large unilamellar vesicles (200 nm) composed of palmitoyl‐2‐oleoyl‐*sn*‐glycero‐3‐phosphocholine (POPC) were prepared with an internal solution containing 100 mM NaCl and 1 mM HPTS, buffered with HEPES to pH 7.0 (Figure 2, see Supporting Information for full experimental details). The vesicles were then suspended in an external solution containing the same NaCl/HEPES buffer but without HPTS. A pH gradient was generated for each experiment by addition of a NaOH base pulse to the extravesicular environment at *t* = −30 s, before the transporter was added as a DMSO solution at *t* = 0 s. Real‐time measurement of the relative fluorescence intensity of both ionisation forms of HPTS (F_460_/F_403_) was used to determine the change in the intravesicular pH induced by either H^+^/Cl^−^ symport or functionally equivalent OH^−^/Cl^−^ antiport mechanisms. Given that aryl squaramides are well‐established chloride binding motifs, and require modification with crown ethers to bind and transport metal cations, Na^+^/H^+^ antiport was not considered a feasible mechanism in this assay [5, 8, 19]. The detergent Triton X‐100 was added at *t* = 210 s to lyse the vesicles and calibrate the 100% Cl^−^ efflux reading. Dose response studies were carried out followed by Hill analysis of the Cl^−^ efflux at 200 s to determine the effective concentration of a transporter required to reach 50% efflux (EC_50_, Figure 3).

The most active transporters in the series were found to be non‐targeted compounds **3** and **6** (0.03 and 0.06 mol%, respectively, Table 1). Notably, the morpholine appended compounds exhibited greater transport activity than their *N,N*‐dimethylamine counterparts (**1** > **2**, **4** > **5**). For compounds **7** and **8** 50% efflux was not reached at the highest concentrations tested, so full dose‐response curves could not be obtained.

**FIGURE 4:**
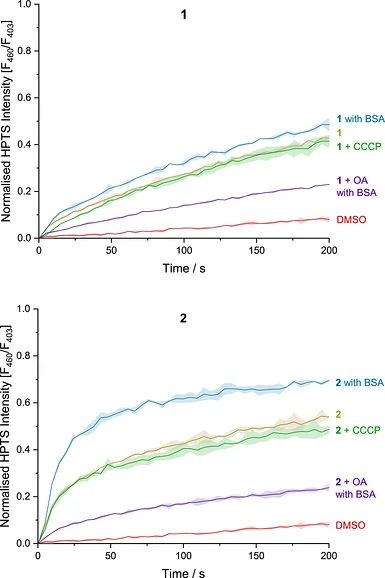
HPTS fluorescence for (a) **1** (0.2 mol%) and (b) **2** (1 mol%) as a free receptor in untreated vesicles (yellow), in the presence of CCCP (1 mol%) (green), in BSA‐treated vesicles (1 mol%) (blue), and in BSA‐treated vesicles in the presence of OA (10 mol%) (purple). A DMSO blank (red) was used as a control.

Transport activity increased with concentration for the anthracene‐containing squaramides **6**–**8** initially, but then appeared to decrease at higher concentrations, with the effect being most pronounced for **7** and **8** (Figures S54,55). We believe this is due to interference of the anthracene fluorophore with the measurement of the HPTS fluorescence intensity (F_460_/F_403_). The anthracene compounds can be excited with 403 nm, which is the wavelength used to excite the protonated form of the HPTS dye. This competitive interference may cause a lower apparent transport reading that does not reflect the actual properties of the transporter. As the concentration of the transporter is lowered the effect appears to decrease. The apparent reduction in transport was minimal for **6**, compared to **7** and **8**, likely due to its weaker emissive properties. Additional transport experiments were performed in the presence of protonophore cyanide‐*m*‐chlorophenylhydrazone (CCCP) to investigate the preference of **1** and **2** for Cl^−^ uniport versus H^+^/Cl^−^ symport mechanisms. In the presence of CCCP, an increase in the rate of transport indicates that proton transport is the rate‐limiting step in the process. This can therefore be used to identify the Cl^−^ > H^+^ transport selectivity. For compounds **1** and **2** minimal change in transport was observed on addition with CCCP (Figure 4), suggesting that both compounds facilitate H^+^/Cl^−^ symport.

Small‐molecule‐based anion transporters have also been shown to independently facilitate proton transport by interacting with free fatty acids, a common contaminant found in commercially available POPC. To investigate any contribution of the fatty acid‐activated mechanism to the observed transport, additional experiments were performed using bovine serum albumin (BSA) to remove fatty acid impurities. Surprisingly, an increase in transport was observed for both **1** and **2** in the absence of free fatty acids (Figure 4, ‘BSA’), though the increase was much greater for compound **2**. Addition of excess oleic acid (OA), a fatty acid, to the BSA‐treated vesicles resulted in diminished transport for both compounds, but again the effect was more pronounced for squaramide **2**. Fatty acid‐activated proton transporters typically show the opposite trend under these conditions, where transport is proportional to the amount of free fatty acids present.

This is evidence in support of the hypothesis that free fatty acid carboxylate groups interact strongly with **1** and **2**, suppressing H^+^/Cl^−^ symport by outcompeting Cl^−^ for transporter binding sites. However, the resultant transporter‐fatty acid complexes do not appear capable of proton transport themselves. This contrasts with reports that structurally related bisaryl squaramides mediate fatty acid‐activated proton transport by shuttling deprotonated fatty acids across the membrane [20]. It is possible that the basic dimethylamine and morpholine groups appended to **1** and **2** favor the formation of polar zwitterionic complexes that are unable to traverse the membrane's hydrophobic interior, preventing transport.

### 2.4 Subcellular Localisation

**FIGURE 5:**
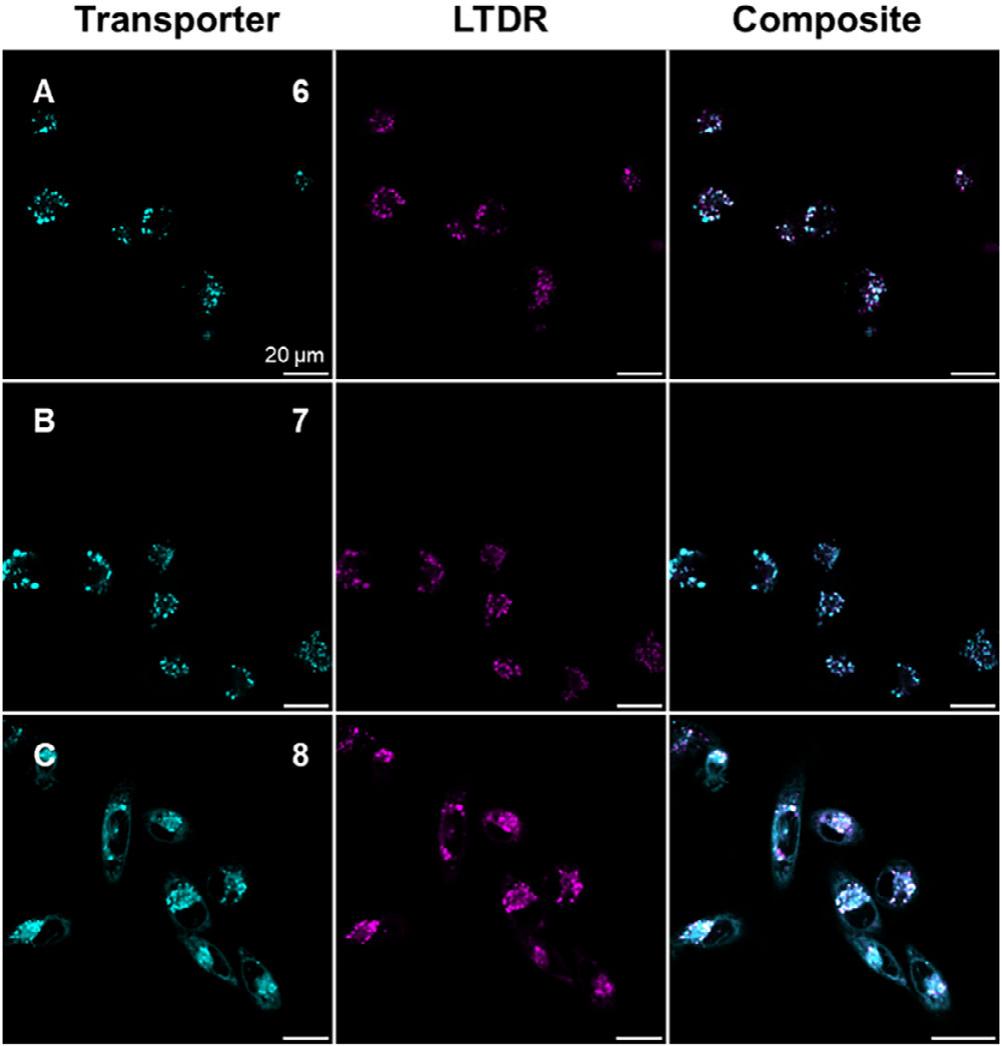
Representative images of live MDA‐MB‐231 breast cancer cells dosed with 5.5 µM of compound **6** (A), **7** (B), and **8** (C) for 3 h, followed by LysoTracker Deep Red (LTDR) (50 nM) for 30 min. Cyan channel (Transporter): *λ*_ex_ = 405 nm, *λ*_em_ = 430 – 600 nm. Magenta channel (LTDR): *λ*_ex_ = 653 nm, *λ*_em_ = 660 – 830 nm. The composite image is also shown. All scale bars represent 20 µm.

To investigate the capacity for the targeted squaramides to accumulate within lysosomes, colocalisation studies were performed using anthracene‐containing compounds **6**–**8**. MDA‐MB‐231 human breast cancer cells (ATCC, RRID: CVCL_0062) were dosed with non‐toxic concentrations of the compounds for 3 h. Colocalisation experiments were carried out with lysosomal stain LysoTracker Deep Red (LTDR) (Figure 5), additionally, Pearson's correlation coefficients (PCC) values were calculated to quantify the degree of colocalisation. For the lysosomal targeted compounds **7** and **8** the PCC values were calculated to be 0.86 and 0.79, respectively, confirming their affinity to localise in the lysosomes. In comparison, the non‐targeted control compound **6** had a much less significant correlation with the lysosomal stain, with a PCC value of 0.52, and exhibited lower fluorescence intensity than the targeted transporters. Comparison of the targeted‐anthracene squaramides showed that cells treated with *N,N*‐dimethylamine‐containing **8** exhibited brighter but more diffuse fluorescence compared to those treated with its morpholine analogue **7**, suggesting increased cellular uptake. There also appeared to be a greater accumulation of LTDR in cells treated with compound **8**.

One possibility is that the *N,N*‐dimethylamine‐appended analogues accumulate more efficiently in lysosomes than their morpholine counterparts, due to differences in their basicities. Using the MolGpKa web server [21, 22], the p*K*_a_ values of the targeting amino group for each of the compounds were calculated. The morpholine‐appended compounds **1**, **4**, and **7** have a calculated p*K*_a_ of 7.1 whereas the *N,N*‐dimethylamine‐appended **2**, **5**, and **8**, have p*K*_a_s of 8.8, 8.9, and 9.1, respectively (Table S1). The higher p*K*_a_ of the *N,N*‐dimethylamine motif suggests that a greater fraction of the transporter is protonated at cytoplasmic pH (7.0–7.5) [23], thereby increasing cellular uptake and subsequently enhancing lysosomal accumulation. It has previously been shown that cationic amphiphilic drugs with weakly basic groups, including *N,N*‐dimethylamine, can hyperaccumulate in lysosomes via this ion trapping mechanism and cause enhanced accumulation of LysoTracker Red by expanding lysosomal volume [24, 25].

Fluorene‐containing compounds **3**, **4**, and **5** could not be used in imaging studies as they do not possess a strong enough fluorescence response at the necessary concentration.

### 2.5 Acridine Orange

**FIGURE 6:**
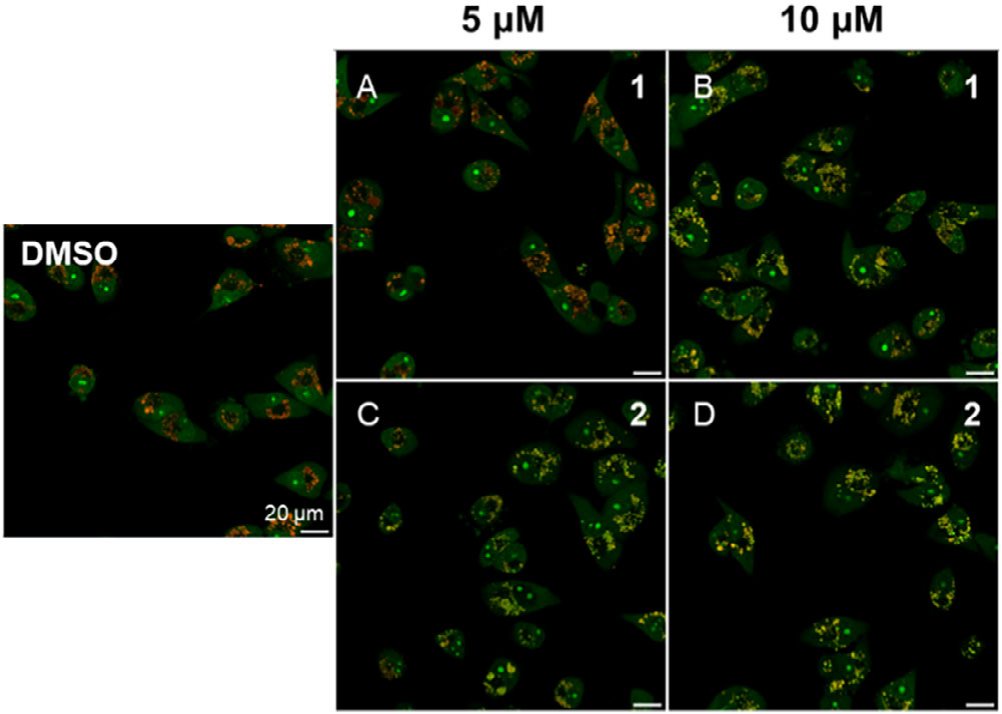
Representative images of live MDA‐MB‐231 breast cancer cells dosed with DMSO, transporter **1 **(A – 5 µM, B – 10 µM) and **2 **(C – 5 µM, D – 10 µM) for 3 h, followed by Acridine Orange (5 µg/mL) for 30 min. All scale bars represent 20 µm.

As the compounds exhibited high lysosome targeting ability, we wanted to explore their ability to deacidify the lysosomes of MDA‐MB‐231 cells. This was done using the pH‐dependent acridine orange (AO) dye. After staining with AO, the cells display a green emission in parts of the cell with higher pH such as the cytosol, and also the nucleus due to the affinity of AO for nucleic acids. The granular orange emission of AO observed is due to protonation of the AO in acidic environments including the lysosomes. In the DMSO control (Figure 6) this orange emission can clearly be seen. However, treatment with transporters **1** and **2** led to a concentration‐dependent reduction in the orange emission, indicative of lysosomal deacidification. This behaviour is consistent with previously studied squaramide‐based H^+^/Cl^−^ transporters, which mediate apoptotic cell death by increasing lysosomal pH and disrupting autophagy [5]. Transporter **2** produced a greater reduction in orange fluorescence than **1** when tested at 5 µM. The stronger ability of *N,N*‐dimethylamine‐appended transporter **2** to deacidify lysosomes could explain its increased cytotoxicity compared to morpholine‐appended transporter **1**, as discussed below.

### 2.6 Cell Viability

The capacity of targeted anionophores **1**–**8** to reduce cell viability was evaluated using an MTS assay in MDA‐MB‐231 breast cancer cells. This triple negative cell line lacks expression of the oestrogen receptor, progesterone receptor, and HER2 expression, and was therefore selected as a clinically aggressive model due to its limited targeted therapies. BEAS‐2B non‐cancerous bronchial cells (ATCC, RRID: CVCL_0168) were used to assess selectivity. Cells were treated with the compounds for 72 h over a wide concentration range and dose‐response curves were used to calculate the half maximum inhibitory concentration (IC_50_, Table 1). Interestingly, in MDA‐MB‐231 cells, the most potent compounds possessed the *N,N*‐dimethyl amine targeting group (**2**, **5**, **8**), with all three compounds exhibiting a cytotoxic effect (IC_50_ = 10.94, 10.68, and 21.80 µM, respectively). On the other hand, the morpholine‐appended **1**, exhibited a weaker antiproliferative profile (IC_50_ = 71.89 µM), despite showing potent activity in the HPTS assay. The remaining compounds, morpholine‐appended **4** and **7**, and non‐targeted **3** and **6**, were not cytotoxic up to their solubility limits, highlighting the importance of the *N,N‐*dimethylamine targeting group. In BEAS‐2B cells the same trends were observed, where the *N,N*‐dimethylamine appended compounds (**2**, **5**, **8**) demonstrated the greatest antiproliferative effect (IC_50_ = 12.55, 10.31, and, 11.17 µM, respectively), compound **1** had a weaker cytotoxic effect (IC_50_ = 20.20 µM), and the remaining compounds demonstrated no cytotoxicity. Overall, the targeted‐squaramides showed no selectivity towards the cancerous cell line, with compounds **1** and **8** exhibiting higher potency in the non‐cancerous BEAS‐2B cells.

**FIGURE 7:**
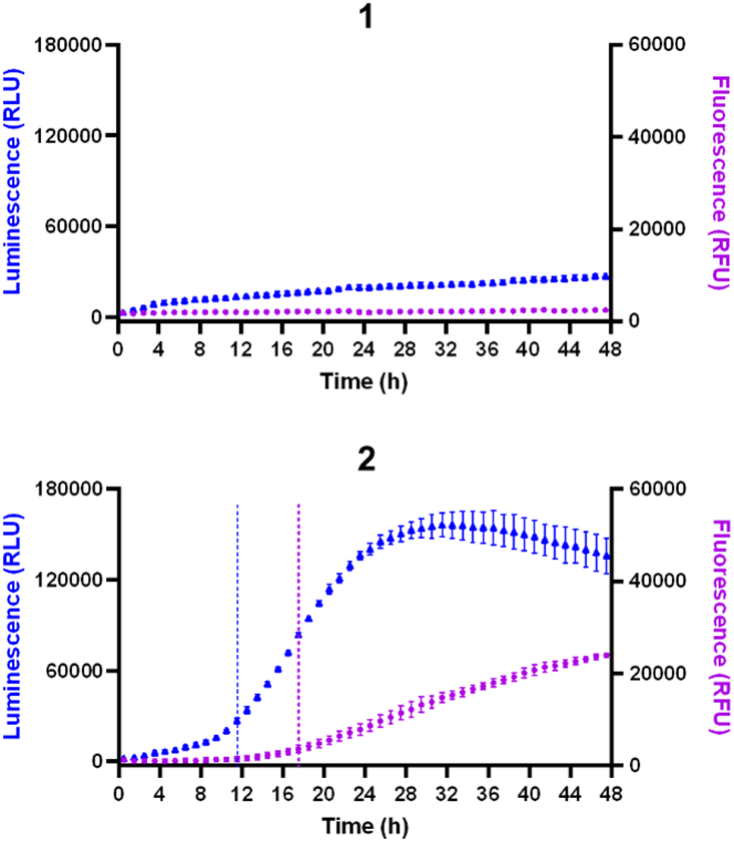
Detection of apoptosis in MDA‐MB‐231 cells treated with **1** and **2** (10 µM) using the RealTime‐Glo Annexin V Apoptosis and Necrosis Assay (Promega). Luminescence and fluorescence signals were monitored over time to detect early apoptotic (phosphatidylserine exposure, blue) and necrotic (membrane compromise, purple) events, respectively. Vertical lines indicate the transition into exponential signal growth. Data shown are from one representative experiment; additional repeats (*N* = 3) are included in the Supporting Information.

We then sought to assess the ability of compound **2** to promote apoptosis using a real‐time live‐cell assay that monitors phosphatidylserine externalisation via luminescence and loss of membrane integrity via fluorescence (full details in the Supporting Information). Treatment with compound **2** (10 µM) produced a luminescent response at ∼12 h followed by a fluorescent response at ∼18 h (Figure 7). This sequence reflects phosphatidylserine externalisation occurring before membrane compromise, which is characteristic of apoptosis [26] and consistent with the behavior of the pro‐apoptotic kinase inhibitor sorafenib (50 µM, Figure S59) [27]. By contrast, compound **1** (10 µM) had no effect under the same conditions.

The cell viability results suggest that the choice of lysosomal targeting group plays a key role in the antiproliferative activity of squaramide‐containing transporters. This marked difference in cytotoxicity was not seen for previously reported lysosome targeted urea‐based anionophores, which showed similar potency for analogues bearing the morpholine and dimethylamine targeting groups [7]. This demonstrates that the morpholine group can be incorporated with other chloride transporter scaffolds, but appears to be incompatible with the squaramide motif. The higher potency of the *N,N*‐dimethylamine‐appended transporters may be due to the observed enhanced lysosomal accumulation, caused by the higher p*K*_a_, as more of the transporter is acting in its intended organelle. Similarly, reduced uptake of morpholine‐appended transporters could account for the non‐cytotoxic activity of these analogues and **SA2**. Although the importance of the *N,N*‐dimethylamine group might suggest that it contributes its own cytotoxic effects, its presence in many non‐toxic small molecules points towards a more nuanced explanation [28, 29, 30, 31, 32]. Previous studies with compound **SA1** supported the hypothesis that the cytotoxicity of this compound arises not solely from chloride transport but also from other factors such as lysosomal stress, redox imbalance and autophagy disruption [33]. Hence chloride transport is expected to be one amongst a series of factors that result in cytotoxicity when cells are exposed to the new targeted‐squaramides reported here.

Overall, targeted squaramides **2** and **5** reduced MDA‐MB‐231 cell viability with the greatest potency, exhibiting IC_50_ concentrations approaching 10 µM, but were slightly less effective than structurally related but non‐targeted bisaryl squaramides (IC_50_ values 4–7 µM) [20]. Thus, lysosomal localisation did not translate into enhanced cytotoxicity for this scaffold, which may suggest that non‐lysosomal pathways contribute to their cytotoxicity, or that the targeting group inadvertently causes off‐target interactions, such as formation of strong zwitterionic fatty acid complexes that impede transport activity. The efficacy of lysosomal targeting against MDA‐MB‐231 cells varies significantly across drug classes. For example, attaching targeting groups to the Hsp90 inhibitor geldanamycin was found to boost its cytotoxicity by fourfold, reducing its IC_50_ concentration from 17 µM to *ca*. 4 µM for both the morpholine and dimethylamine appended analogues [34]. In contrast, when a similar approach was employed to enhance lysosomal cathepsin D release by ruthenium(II) *p*‐cymene complexes, the presence of morpholine targeting groups resulted in similar or inferior activity, with IC_50_ values between 1–5 µM [35]. Thus, while lysosomal targeting remains a promising strategy, its benefits are not universal and were found in this study to vary significantly between different chloride transporters. Future work should consider that the choice of targeting group can heavily influence overall cytotoxicity, and that the group's compatibility with the transport motif employed must be evaluated on a scaffold‐by‐scaffold basis.

## 3 Conclusions

A new family of lysosome‐targeted anion transporters were synthesised using a central squaramide scaffold. Binding studies demonstrated that distancing the lysosomal targeting groups from the binding motif via a 1,4‐substituted benzene improved Cl^−^ binding affinity compared to **SA2** and **SA3**. Despite this, vesicular chloride transport studies showed that incorporation of the targeting groups diminished transport activity with respect to non‐targeted control **3** and **6**. Among the targeted transporters, those appended with the morpholine group exhibited greater transport activity than the *N,N*‐dimethylamine analogues. However, cell viability studies revealed that the *N,N*‐dimethylamine‐appended analogues (**2**, **5**, **8**) were significantly more cytotoxic than both their morpholine and non‐targeted counterparts, despite their lower anion transport activity, and were found to induce apoptotic cell death. Unlike previous lysosome‐targeted chloride transporters, these findings suggest that the cytotoxicity of lysosome‐targeted squaramides depends heavily on the properties of the targeting motif employed. Subcellular colocalisation studies confirmed that both targeting groups directed the squaramides to the lysosome. However, the dimethylamine‐appended analogue (**8**) exhibited greater lysosomal accumulation, likely due to the higher p*K*_a_ of this motif, which promotes retention in the lysosome's acidic environment by ion trapping, providing a possible explanation for its increased cytotoxicity.

## Conflicts of Interest

The authors declare no conflicts of interest.

## Supporting information



The authors have cited additional references within the Supporting Information [36, 37].

## Data Availability

The data that support the findings of this study are available in the supplementary material of this article.
