# Supplementary material for: Lysosome‐Targeted Squaramide Anion Transporters
Source: Chemistry. 2026 Jun 9;32(36):e71243. doi: 10.1002/chem.71243 (PMC13602887; doi:10.1002/chem.71243)
Supplement: Supplementary file 1 [file CHEM-32-e71243-s001.docx]

Lysosome-Targeted Squaramide Anion Transporters

Elba Feo,^[a]^ Edward York,^[a, d]^ Anthony Thai,^[a]^ William G. Ryder,^[b]^ Daniel A. McNaughton,^[c]^ and Philip A. Gale.*^[a,d]^

^[a]^*School of Mathematical and Physical Sciences, Faculty of Science, University of Technology Sydney, Sydney, NSW 2007, Australia.*

^[b]^*School of Chemistry, The University of Sydney, Sydney, NSW, 2006*

^[c^*Monash Institute of Pharmaceutical Science Monash Institute of Pharmaceutical Science, Monash University, Parkville, VIC 3052, Australia*

^[d]^*University of Groningen, Faculty of Science and Engineering, Zernike Institute for Advance Materials, Nijenborgh 3, 9747 AG Groningen, The Netherlands.*

** E-mail:* [*p.a.gale@rug.nl*](mailto:p.a.gale@rug.nl)

Contents

[1. General Remarks	3](#_Toc216879070)

[2. Synthesis	3](#_Toc216879071)

[3. Characterisation Data	11](#_Toc216879072)

[4. ^1^H NMR Titration Experiments	23](#_Toc216879073)

[4.1. Sample Preparation	23](#_Toc216879074)

[4.2. Titration Procedure	23](#_Toc216879075)

[4.3. Titration Data Fitting	23](#_Toc216879076)

[4.4 Titration Data with TBACl Guest	24](#_Toc216879077)

[5. HPTS Assay	33](#_Toc216879078)

[5.1. Vesicle Preparation	33](#_Toc216879079)

[5.2. HPTS Assay Hill Analyses	33](#_Toc216879080)

[5.3. HPTS Assay BSA Mechanistic Studies	37](#_Toc216879081)

[6. pKa Data	38](#_Toc216879082)

[7. Photophysical Studies	38](#_Toc216879083)

[8. Cell Culture	39](#_Toc216879084)

[8.1. Cell Culture Conditions	39](#_Toc216879085)

[8.2. MTS Cell Viability Assay	39](#_Toc216879086)

[8.3. Apoptosis and Necrosis Assay	40](#_Toc216879087)

[9. Confocal Microscopy	41](#_Toc216879088)

[9.1. Colocalisation Imaging	41](#_Toc216879089)

[9.2. Leica Stellaris 8 Confocal Microscope	41](#_Toc216879090)

[9.3. Image Data Analysis	42](#_Toc216879091)

# 1. General Remarks

Unless otherwise stated, all chemicals and solvents were purchased from commercial suppliers and used without further purification. Deionised water was collected from a Merck Millipore Milli-Q™ reference ultrapure water purification system.

NMR spectra were collected on a Bruker Ascend 400 spectrometer. Chemical shifts (δ) were reported in ppm and calibrated using the residual solvent peak of DMSO-*d_6._* Data was reported with spin multiplicities abbreviated to s = singlet, d = doublet, t = triplet, q = quartet, and m = multiplet, coupling constant *J* in Hz, and the relative integral.

High-resolution mass spectra (HR-MS) were recorded on an Agilent Technologies 6510 Q-TOF LCMS.

HPTS transport data was collected on an Agilent Cary Eclipse Fluorescence Spectrophotometer equipped with a temperature-controlled multicell holder (25 °C). MTS assay and RealTime-Glo^TM^ Annexin V Apoptosis and Necrosis Assay were performed using a Tecan Spark plate reader.

# 2. Synthesis

#### 3-ethoxy-4-[[4-(trifluoromethyl)phenyl]amino]-3-cyclobutene-1,2-dione (**9**)

3, 5-bis(trifluoromethyl)aniline (0.68 mL, 4.37 mmol) was added to a stirred solution of diethyl squarate (0.71 mL, 4.80 mmol) and zinc trifluoromethanesulfonate (349 mg, 0.96 mmol) in ethanol (15 mL). The solution was stirred at room temperature for 24 hours. A pale-yellow precipitate formed, which was isolated by filtration and washed with ethanol (3 x 5 mL) to afford the product (1.047 g, 2.96 mmol, 68 %).

**^1^H NMR**: (400 MHz, DMSO-*d_6_*) δ (ppm): 11.21 (1 H, s), 8.04 (2 H, s), 7.78 (1 H, s), 4.81 (2 H, q, *J =* 7.1), 1.42 (3 H, t, *J* *=* 7.1).

**^13^C NMR:** (101 MHz, DMSO-*d_6_*) δ (ppm):  187.9, 185.0, 179.7, 169.7, 140.7, 131.6 (q, *J_C-F_ =* 33.1), 123.6 (q, *J_C-F_ =* 272.8), 119.5, 116.8, 70.6, 15.8.

**HR-MS** (ESI^+^) calcd for C_14_H_9_F_6_NO_3_ [M + H]^+^: 354.05594 found *m*/*z* 354.05322.

N-(3,5-bis(trifluoromethyl)phenyl)-N′-(ethyl-N′′-morpholine)-squaramide (**SA2**)

4-(2-aminoethyl)morpholine (120 mg, 0.92 mmol) was added to a stirred solution of **9** (250 mg, 0.71 mmol) and zinc trifluoromethanesulfonate (129 mg, 0.36 mmol) in MeOH (15 mL). The solution was stirred at 70 °C for 24 hours. An off-white precipitate formed, which was isolated by filtration and washed with MeOH (3 x 10 mL) to afford the product (137 mg, 0.31 mmol, 44 %).

**^1^H NMR**: (400 MHz, DMSO-*d_6_*) δ (ppm): 10.32 (1 H, s), 8.04 (2 H, s), 7.66 (1 H, s), 3.74 (2 H, s), 3.58 (1 H, s), 2.43 (4 H, s) in agreement with literature values.^[9]^

N-(3,5-bis(trifluoromethyl)phenyl)-N′-(ethyl-N′′,N’’-dimethylamine)-squaramide (**SA3**)

2-dimethylaminoethylamine (69 mg, 0.78 mmol) was added to a stirred solution of **9** (250 mg, 0.71 mmol) and zinc trifluoromethanesulfonate (129 mg, 0.36 mmol) in MeOH (15 mL). The solution was stirred at 70 °C for 24 hours. A white precipitate formed, which was isolated by filtration and washed with MeOH (3 x 10 mL) to afford the product (130 mg, 0.33 mmol, 46 %).

**^1^H NMR**: (400 MHz, DMSO-*d_6_*) δ (ppm): 10.34 (1 H, s), 8.05 (2 H, s), 7.77 (1 H, s), 7.66 – 7.60 (1 H, m), 3.72 (2 H, d, *J* = 6.1), 2.47 (2 H, t, *J* = 5.8), 2.22 (5 H, s).

**^13^C NMR:** (101 MHz, DMSO-*d_6_*) δ (ppm): 185.29, 180.74, 170.02, 162.69, 141.74, 131.81 (q, *J_C-F_ =* 33.3), 123.64 (q, *J_C-F_ =* 272.8), 118.33, 115.00, 59.22, 45.47, 41.95.

**HR-MS** (ESI^+^) calcd for C_16_H_15_F_6_N_3_O_2_ [M + H]^+^: 396.11412 found *m*/*z*  396.11683.

#### 3-ethoxy-4-(2-aminofluorene)-3-cyclobutene-1,2-dione (**10**)

2-aminofluorene (320 mg, 1.76 mmol) was added to a stirred solution of diethyl squarate (0.24 mL, 1.60 mmol) and zinc trifluoromethanesulfonate (291 mg, 0.80 mmol) in ethanol (15 mL). The solution was stirred at room temperature for 24 hours. A pale-yellow precipitate formed, which was isolated by filtration and washed with ethanol (3 x 5 mL) to afford the product (484 mg, 1.59 mmol, 98 %).

**^1^H NMR**: (400 MHz, DMSO-*d_6_*) δ (ppm): 10.88 (1H, s), 7.85 (2H, t, *J* = 7.7 Hz), 7.60 (1H, s), 7.56 (1H, d, *J* = 7.4 Hz), 7.41 (1H, s), 7.37 (1H, t, *J* = 7.3 Hz), 7.29 (2H, td, *J* = 7.4, 1.0 Hz), 4.79 (2H, q, *J* = 7.1 Hz), 3.92 (2H, s), 3.34 (H2O, s), 1.44 (3H, t, *J* = 7.1 Hz).

**^13^C NMR:** (101 MHz, DMSO-*d_6_*) δ (ppm):  184.1, 169.8, 144.5, 143.3, 141.1, 137.7, 137.4, 127.3, 126.9, 125.5, 120.9, 120.2, 119.0, 116.9, 70.0, 37.0, 16.1.

**HR-MS** (ESI^+^) calcd for C_19_H_15_NO_3_ [M + H]^+^: 306.11247 found *m*/*z* 306.11397.

#### 4-(2-N-morpholinoethyl)aniline (**11**)

Morpholine (1 mL, 11.59 mmol) was added to 1-(2-bromoethyl)-4-nitrobenzene (500 mg, 2.18 mmol) in DCM (10 mL) with triethylamine (0.5 mL). The reaction was stirred at room temperature for 72 hours. Water was added and the organic phase was separated. The organic phase was washed with water (x3) and brine, dried over Na_2_SO_4_, and concentrated *in vacuo* to afford an orange oil. The oil was dissolved in EtOH (20 mL) and purged with nitrogen. Catalytic palladium on carbon was added, followed by dropwise addition of hydrazine hydrate (1 mL, 19.98 mmol). The reaction was stirred at 60 °C under N_2_ for 2 hours. The mixture was filtered through celite, washed with ethanol, and concentrated *in vacuo.* The residue was dissolved in EtOAc, washed with water, dried over Na_2_SO_4_, and concentrated *in vacuo* to afford the product as a white solid (307 mg, 1.49 mmol, 68 %).

**^1^H NMR**: (400 MHz, DMSO-*d_6_*) δ (ppm): 6.85 (2 H, d, *J =* 8.4), 6.48 (2 H, d, *J =* 8.4), 4.81 (2 H, s), 3.57 (4 H, t, *J =* 4.7), 2.59 – 2.52 (2 H, m), 2.44 – 2.35 (6 H, m).

**^13^C NMR:** (101 MHz, DMSO-*d_6_*) δ (ppm):  147.0, 129.4, 127.6, 114.4, 66.7, 61.3, 53.8, 32.2.

**HR-MS** (ESI^+^) calcd for C_12_H_18_N_2_O [M + H]^+^: 207.14919 found *m*/*z* 207.14912.

#### 4-(2-dimethylaminoethyl)nitrophenyl (**12**)

*N,N*-dimethylamine hydrochloride (265 mg, 3.25 mmol) was added to 1-(2-bromoethyl)-4-nitrobenzene (250 mg, 1.09 mmol) in DCM (10 mL) with triethylamine (0.6 mL, 4.30 mmol). The reaction was stirred at room temperature for 72 hours. Water was added and the layers were separated. The organic layer was washed with 10% citric acid twice. The combined aqueous layers were basified with 20% NaOH to bring the pH to 11. The product was extracted with DCM. The organic layer was then washed with brine, dried over Na_2_SO_4_, and concentrated *in vacuo* to afford the product as an orange oil (171 mg, 0.88 mmol, 82 %).

**^1^H NMR**: (400 MHz, CDCl_3_) δ (ppm): 8.13 (2 H, d, *J =* 8.8), 7.36 (2 H, d, *J =* 8.8), 2.91 – 2.83 (2 H, m), 2.56 (2 H, dd, *J =* 8.7, 6.8), 2.28 (6 H, s).

**^13^C NMR:** (101 MHz, CDCl_3_) δ (ppm): 148.5, 146.6, 129.6, 123.8, 60.7, 45.5, 34.2.

**HR-MS** (ESI^+^) calcd for C_10_H_14_N_2_O_2_ [M + H]^+^: 195.13862 found *m*/*z* 195.11288.

#### 4-(2-dimethylaminoethyl)aniline (**13**)

**11** (171 mg, 0.88 mmol) was dissolved in EtOH (20 mL) and purged with nitrogen. Catalytic palladium on carbon was added, followed by dropwise addition of hydrazine hydrate (1 mL, 19.98 mmol). The reaction was stirred at 60 °C under N_2_ for 2 hours. The mixture was filtered through celite, washed with ethanol, and concentrated *in vacuo.* The residue was dissolved in EtOAc, washed with water, dried over Na_2_SO_4_, and concentrated *in vacuo* to afford the product as a white solid (130 mg, 0.79 mmol, 90 %).

**^1^H NMR**: (400 MHz, CDCl_3_) δ (ppm): 6.99 (2 H, d, *J =* 8.4), 6.62 (2 H, d, *J =* 8.4), 3.55 (2 H, s), 2.71 – 2.62 (2 H, m), 2.51 – 2.43 (2 H, m), 2.28 (6 H, s).

**^13^C NMR:** (101 MHz, CDCl_3_) δ (ppm): 144.5, 130.5, 129.5, 115.4, 62.0, 45.6, 33.6.

**HR-MS** (ESI^+^) calcd for C_10_H_16_N_2_ [M + H]^+^: 165.13862 found *m*/*z* 165.13890.

N-(3,5-bis(trifluoromethyl)phenyl)-N′-(4-phenylethyl-N′′-morpholine)-squaramide (**1**)

**11** (96 mg, 0.47 mmol) was added to a stirred solution of **9** (150 mg, 0.42 mmol) and zinc trifluoromethanesulfonate (76 mg, 0.21 mmol) in MeOH (15 mL). The solution was stirred at 70 °C for 24 hours. An off-white precipitate formed, which was isolated by filtration and washed with MeOH (3 x 10 mL) to afford the product (112 mg, 0.22 mmol, 52 %).

**^1^H NMR**: (400 MHz, DMSO-*d_6_*) δ (ppm): 10.31 (1 H, s), 10.02 (1 H, s), 8.03 (2 H, s), 7.71 (1 H, s), 7.33 (2 H, d, *J* = 8.0), 7.23 (2 H, d, *J* = 8.0), 3.58 (4 H, t, *J* = 4.5), 2.71 (2 H, t, *J* = 7.9), 2.49 – 2.40 (4 H, m).

**^13^C NMR:** (101 MHz, DMSO-*d_6_*) δ (ppm): 183.5, 182.7, 167.4, 165.1, 141.6, 136.7, 131.9 (q, *J_C-F_* 33.1), 129.8, 127.7, 125.0, 122.3, 119.6, 119.2, 115.6, 66.7, 60.3, 53.8, 32.3.

**HR-MS** (ESI^+^) calcd for C_24_H_21_F_6_N_3_O_3_ [M + H]^+^: 514.15599 found *m*/*z* 514.15867.

**MP:** 207–209 °C

N-(3,5-bis(trifluoromethyl)phenyl)-N′-(4-phenylethyl-N′′,N′′-dimethylamine)-squaramide (**2**)

**13** (83 mg, 0.51 mmol) was added to a stirred solution of **9** (150 mg, 0.42 mmol) and zinc trifluoromethanesulfonate (76 mg, 0.21 mmol) in MeOH (15 mL). The solution was stirred at 70 °C for 24 hours. A precipitate formed, which was isolated by filtration and washed with MeOH (3 x 10 mL). The precipitate was resuspended in EtOAc, TEA (1 mL) was added, and the suspension was stirred for 5 mins. The solvent was then removed *in vacuo*. The residue was washed with water and MeOH to afford the product as a pale pink precipitate (55 mg, 0.12 mmol, 28 %).

**^1^H NMR**: (400 MHz, DMSO-*d_6_*) δ (ppm): 10.07 (2 H, s), 8.03 (2 H, s), 7.69 (1 H, s), 7.35 (2 H, d, *J* = 8.0), 7.23 (2 H, d, *J* = 8.0), 2.72 (2 H, t, *J* = 7.6), 2.60 (2 H, d, *J* = 7.9), 2.31 (6 H, s).

**^13^C NMR:** (101 MHz, DMSO-*d_6_*) δ (ppm): 182.7, 182.0, 166.6, 164.4, 140.9, 136.0, 135.2, 131.0 (q, *J_C-F_* = 33.2), 129.0, 126.9, 124.2, 121.5, 119.7, 118.8, 118.4, 114.7, 59.8, 44.2, 31.7.

**HR-MS** (ESI^+^) calcd for C_22_H_19_F_6_N_3_O_2_ [M + H]^+^: 472.14542 found *m*/*z* 472.14950.

**MP:** 149–151 °C

N-(3,5-bis(trifluoromethyl)phenyl)-N′-(2-fluorene)-squaramide (**3**)

2-aminofluorene (113 mg, 623 μmol) was added to a stirred solution of **9** (200 mg, 566 μmol) and zinc trifluoromethanesulfonate (103 mg, 283 μmol) in ethanol (10 mL). The solution was stirred at 65 °C for 24 hours. A yellow precipitate formed, which was isolated by filtration and washed with methanol (3 x 10 mL) to afford the product (235 mg, 480 μmol, 85 %).

**^1^H NMR**: (400 MHz, DMSO-*d_6_*) δ (ppm): 10.30 (2 H, s), 8.05 (2 H, s), 7.86 (2 H, dd, *J* = 14.1, 7.9), 7.69 (2 H, d, *J* = 7.6), 7.57 (1 H, d, *J* = 7.4), 7.45 – 7.33 (2 H, m), 7.29 (1 H, td, *J* = 7.4, 1.2), 3.93 (2 H, s).

**^13^C NMR:** (101 MHz, DMSO-*d_6_*) δ (ppm): 183.6, 182.9, 167.3, 165.3, 144.9, 143.3, 141.6, 141.2, 137.7, 137.6, 131.9 (q, *J_C-F_* = 32.9)., 127.7, 127.3, 126.8, 125.4, 125.0, 122.3, 121.0, 120.0, 119.3, 119.3, 118.6, 116.5, 115.7, 37.1.

**HR-MS** (ESI^+^) calcd for C_25_H_14_F_6_N_2_O_2_ [M + H]^+^: 489.10322 found *m*/*z* 489.10183.

**MP:** 246–248 °C

N-(2-fluorene)-N′-(4-phenylethyl-N′′-morpholine)-squaramide (**4**)

**11** (56 mg, 0.27 mmol) was added to a stirred solution of **10** (87 mg, 0.25 mmol) and zinc trifluoromethanesulfonate (45 mg, 0.12 mmol) in toluene:DMF (19:1) (10 mL). The solution was stirred at 70 °C for 24 hours. A pale orange precipitate formed, which was isolated by filtration and washed with MeOH (3 x 10 mL) to afford the product. (82 mg, 0.18 mmol, 72 %).

**^1^H NMR**: (400 MHz, DMSO-*d_6_*) δ (ppm): 9.98 (1 H, s), 9.87 (1 H, s), 7.86 (2 H, dd, *J =* 15.5, 7.9), 7.74 (1 H, s), 7.56 (1 H, d, *J =* 7.5), 7.46 (1 H, dd, *J =* 8.2, 2.1), 7.42 – 7.33 (3 H, m), 7.28 (1 H, dd, *J =* 8.0, 6.9), 7.23 (2 H, d, *J =* 8.3), 3.93 (2 H, s), 3.57 (4 H, t, *J =* 4.6), 2.74 – 2.65 (2 H, m), 2.49 – 2.36 (6 H, m).

**^13^C NMR:** (101 MHz, DMSO-*d_6_*) δ (ppm):  δ 181.6, 165.6, 165.2, 144.5, 142.8, 140.7, 137.7, 136.5, 136.5, 135.5, 129.5, 126.8, 126.2, 125.1, 120.7, 119.6, 118.6, 117.5, 115.3, 66.2, 60.1, 53.3, 36.6, 31.7.

**HR-MS** (ESI^+^) calcd for C_29_H_27_N_3_O_3_ [M + H]^+^: 466.21252 found *m*/*z* 466.20971.

**MP:** 234–236 °C

N-(2-fluorene)-N′-(4-phenylethyl-N′′,N′′-dimethylamine)-squaramide (**5**)

**13** (60 mg, 0.36 mmol) was added to a stirred solution of **10** (115 mg, 0.33 mmol) and zinc trifluoromethanesulfonate (60 mg) in toluene:DMF (19:1) (10 mL). The solution was stirred at 70 °C for 24 hours. A precipitate formed, which was isolated by filtration and washed with MeOH (3 x 10 mL). The precipitate was resuspended in EtOAc, TEA (1 mL) was added, and the suspension was stirred for 5 mins. The solvent was then removed *in vacuo*. The residue was washed with water and MeOH to afford the product as a cream solid (101 mg, 0.24 mmol, 73 %).

**^1^H NMR**: (400 MHz, DMSO-*d_6_*) δ (ppm): 10.30 (1 H, s), 10.20 (1 H, s), 8.19 (2 H, dd, *J* = 16.0, 7.9), 8.07 (1 H, s), 7.89 (1 H, d, *J =* 7.4), 7.79 (1 H, d, *J* = 8.2), 7.77 – 7.66 (3 H, m), 7.59 (3 H, dd, *J* = 20.0, 7.7), 4.26 (2 H, s), 3.10 – 3.02 (2 H, m), 2.97 (2 H, s).

**^13^C NMR:** (101 MHz, DMSO-*d_6_*) δ (ppm): 182.3, 166.3, 166.0, 144.9, 143.3, 141.3, 138.2, 137.3, 137.2, 135.2, 129.9, 127.2, 126.7, 125.4, 121.0, 120.0, 119.3, 118.1, 116.0, 60.3, 44.9, 44.8, 37.1, 32.2.

**HR-MS** (ESI^+^) calcd for C_27_H_25_N_3_O_2_ [M + H]^+^: 424.20195 found *m*/*z* 424.20360.

**MP:** 229–231 °C

N-(3,5-bis(trifluoromethyl)phenyl)-N′-(2-anthracene)-squaramide (**6**)

2-aminoanthracene (82 mg, 0.42 mmol) was added to a stirred solution of **9** (150 mg, 0.42 mmol) and zinc trifluoromethanesulfonate (76 mg, 0.21 mmol) in toluene:DMF (19:1) (15 mL). The solution was stirred at 70 °C for 24 hours. A precipitate formed, which was isolated by filtration and washed with MeOH (3 x 10 mL) to afford the product as a green solid (138 mg, 0.27 mmol, 65 %).

**^1^H NMR**: (400 MHz, DMSO-*d_6_*) δ (ppm): 10.39 (2 H, s), 8.52 (1 H, s), 8.44 (1 H, s), 8.12 (1 H, d, *J =* 9.3), 8.08 – 7.97 (5 H, m), 7.68 – 7.60 (2 H, m), 7.48 (2 H, p, *J =* 6.6).

**^13^C NMR:** (101 MHz, DMSO-*d_6_*) δ (ppm): 183.7, 183.0, 167.1, 165.6, 141.3, 135.9, 132.5, 131.9, 131.8 (q, *J_C-F_* = 33.0), 131.2, 130.2, 129.0, 128.6, 128.1, 126.6, 126.3, 125.6, 125.4, 125.0, 122.2, 120.6, 119.3, 115.9, 114.5.

**HR-MS** (ESI^+^) calcd for C_26_H_14_F_6_N_2_O_2_ [M + H]^+^: 501.10322 found *m*/*z* 501.10642.

**MP:** 258–259 °C

#### 3-ethoxy-4-(2-aminoanthracene)-3-cyclobutene-1,2-dione (**14**)

2-aminoanthracene (100 mg, 0.52 mmol) was added to a stirred solution of diethyl squarate (0.085 mL, 0.57 mmol) and zinc trifluoromethanesulfonate (40 mg, 0.11 mmol) in EtOH (10 mL). The solution was stirred at room temperature for 24 hours. A precipitate formed, which was isolated by filtration and washed with ethanol (3 x 10 mL) to afford the product as an ochre solid (160 mg, 0.50 mmol, 97 %).

**^1^H NMR**: (400 MHz, DMSO-*d_6_*) δ (ppm): 11.02 (1 H, s), 8.54 (1 H, s), 8.45 (1 H, s), 8.14 – 8.03 (3 H, m), 8.00 (1 H, s), 7.62 (1 H, d, *J* = 9.2), 7.50 (2 H, p, *J =* 6.4), 4.84 (2 H, q, *J* = 7.1), 3.47 (8 H, s), 1.48 (3 H, t, *J* = 7.0).

**^13^C NMR:** (101 MHz, DMSO-*d_6_*) δ (ppm): 196.4, 184.0, 169.5, 135.2, 131.9, 131.2, 130.7, 129.4, 128.5, 128.1, 127.7, 126.2, 125.9, 125.2, 125.1, 120.6, 114.6, 69.7, 15.7.

**HR-MS** (ESI^+^) calcd for C_20_H_15_NO_3_ [M + H]^+^: 318.11247 found *m*/*z* 318.11950.

N-(2-anthracene)-N′-(4-phenylethyl-N′′-morpholine)-squaramide (**7**)

**11** (65 mg, 32 mmol) was added to a stirred solution of **14** (100 mg, 32 mmol) and zinc trifluoromethanesulfonate (57 mg, 0.16 mmol) in toluene:DMF (19:1) (15 mL). The solution was stirred at 70 °C for 24 hours. A precipitate formed, which was isolated by filtration and washed with MeOH (3 x 10 mL) to afford the product as a yellow solid (97 mg, 0.20 mmol, 64 %).

**^1^H NMR**: (400 MHz, DMSO-d_6_) δ (ppm): 10.15 (1 H, s), 9.96 (1 H, s), 8.55 (1 H, s), 8.47 (1 H, s), 8.17 – 8.10 (2 H, m), 8.07 (2 H, t, *J* = 6.9), 7.71 (1 H, d, *J* = 9.0), 7.55 – 7.45 (2 H, m), 7.43 (2 H, d, *J* = 8.0), 7.25 (2 H, d, *J* = 8.0), 3.59 (4 H, s), 2.72 (2 H, t, *J* = 8.0), 2.43 (6 H, s).

**^13^C NMR:** (101 MHz, DMSO-*d_6_*) δ (ppm): 182.7, 182.4, 166.7, 165.9, 137.0, 136.4, 136.3, 132.6, 132.1, 131.1, 130.2, 129.9, 128.9, 128.6, 128.1, 126.6, 126.3, 125.5, 125.3, 120.6, 119.3, 113.7, 66.7, 60.3, 53.8, 32.3.

**HR-MS** (ESI^+^) calcd for C_30_H_27_N_3_O_3_ [M + H]^+^: 478.215252 found *m*/*z* 478.21302.

**MP:** 271–272 °C

N-(2-anthracene)-N′-(4-phenylethyl-N′′,N′′-dimethylamine)-squaramide (**8**)

**13** (78 mg, 47 mmol) was added to a stirred solution of **14** (150 mg, 474 mmol) and zinc trifluoromethanesulfonate (86 mg, 0.24 mmol) in toluene:DMF (19:1) (15 mL). The solution was stirred at 70 °C for 24 hours. A precipitate formed, which was isolated by filtration and washed with MeOH (3 x 10 mL) to afford the product as a yellow solid (125 mg, 0.29 mmol, 61 %).

**^1^H NMR**: (400 MHz, DMSO-d_6_) δ (ppm): 10.16 (1 H, s), 9.98 (1 H, s), 8.55 (1 H, s), 8.47 (1 H, s), 8.17 – 8.10 (2 H, m), 8.10 – 8.04 (2 H, m), 7.72 (1 H, dd, *J* = 9.0, 2.3), 7.55 – 7.42 (4 H, m), 7.28 (2 H, d, *J* = 8.2), 2.97 – 2.87 (2 H, m), 2.83 (2 H, dd, *J* = 9.8, 5.5), 2.56 (6 H, s).

**^13^C NMR:** (101 MHz, DMSO-d_6_) δ (ppm):  182.5, 166.6, 165.8, 136.9, 136.3, 132.4, 132.0, 131.0, 130.3, 130.0, 128.8, 128.6, 128.2, 126.7, 126.4, 125.5, 125.3, 120.6, 119.2, 113.4, 61.3, 45.6, 33.1.

**HR-MS** (ESI^+^) calcd for C_28_H_25_N_3_O_2_ [M + H]^+^: 436.20195 found *m*/*z* 436.20202.

**MP:** 269–271 °C

# 3. Characterisation Data

N-(3,5-bis(trifluoromethyl)phenyl)-N′-(ethyl-N′′,N’’-dimethylamine)-squaramide (**SA3**)

**Figure S1.** ^1^H NMR (400 MHz) spectrum of **1** in DMSO-*d_6_* at 298 K.

**Figure S2.** ^13^C NMR (101 MHz) spectrum of **1** in DMSO-*d_6_* at 348.15 K.

**Figure S3.** HR-MS (ESI^+^) spectrum of *N-(3,5-bis(trifluoromethyl)phenyl)-N′-(ethyl-N′′,N’’-dimethylamine)-squaramide (****SA3****)*

N-(3,5-bis(trifluoromethyl)phenyl)-N′-(4-phenylethyl-N′′-morpholine)-squaramide (**1**)

**Figure S4.** ^1^H NMR (400 MHz) spectrum of **1** in DMSO-*d_6_* at 298 K.

**Figure S5.** ^13^C NMR (101 MHz) spectrum of **1** in DMSO-*d_6_* at 348.15 K.

**Figure S6.** HR-MS (ESI^+^) spectrum of *N*-(3,5-bis(trifluoromethyl)phenyl)-*N′*-(4-phenylethyl-*N′′*-morpholine)-squaramide (**1**)

N-(3,5-bis(trifluoromethyl)phenyl)-N′-(4-phenylethyl-N′′,N′′-dimethylamine)-squaramide (**2**)

**Figure S7.** ^1^H NMR (400 MHz) spectrum of **2** in DMSO-*d_6_* at 298 K.

**Figure S8.** ^13^C NMR (101 MHz) spectrum of **2** in DMSO-*d_6_* at 348.15 K.

**Figure S9.** HR-MS (ESI^+^) spectrum of *N*-(3,5-bis(trifluoromethyl)phenyl)-*N′*-(4-phenylethyl-*N′′,N′′*-dimethylamine)-squaramide (**2**)

N-(3,5-bis(trifluoromethyl)phenyl)-N′-(2-fluorene)-squaramide (**3**)

**Figure S10.** ^1^H NMR (400 MHz) spectrum of **3** in DMSO-*d_6_* at 298 K.

**Figure S11.** ^13^C NMR (101 MHz) spectrum of **3** in DMSO-*d_6_* at 348.15 K.

**Figure S12.** HR-MS (ESI^+^) spectrum of *N*-(3,5-bis(trifluoromethyl)phenyl)-*N′*-(2-fluorene)-squaramide (**3**)

N-(2-fluorene)-N′-(4-phenylethyl-N′′-morpholine)-squaramide (**4**)

**Figure S13.** ^1^H NMR (400 MHz) spectrum of **4** in DMSO-*d_6_* at 298 K.

**Figure S14.** ^13^C NMR (101 MHz) spectrum of **4** in DMSO-*d_6_* at 298 K.

**Figure S15.** HR-MS (ESI^+^) spectrum of *N*-(2-fluorene)-*N′*-(4-phenylethyl-*N′′*-morpholine)-squaramide (**4**)

N-(2-fluorene)-N′-(4-phenylethyl-N′′,N′′-dimethylamine)-squaramide (**5**)

 **Figure S16.** ^1^H NMR (400 MHz) spectrum of **5** in DMSO-*d_6_* at 298 K.

**Figure S17.** ^13^C NMR (101 MHz) spectrum of **5** in DMSO-*d_6_* at 348.15 K.

**Figure S18.** HR-MS (ESI^+^) spectrum of *N*-(2-fluorene)-*N′*-(4-phenylethyl-*N′′,N′′*-dimethylamine)-squaramide (**5**)

N-(3,5-bis(trifluoromethyl)phenyl)-N′-(2-anthracene)-squaramide (**6**)

**Figure S19.** ^1^H NMR (400 MHz) spectrum of **6** in DMSO-*d_6_* at 298 K.

**Figure S20.** ^13^C NMR (101 MHz) spectrum of **6** in DMSO-*d_6_* at 348.15K.

**Figure S21.** HR-MS (ESI^+^) spectrum of *N*-(3,5-bis(trifluoromethyl)phenyl)-*N′*-(2-anthracene)-squaramide (**6**)

N-(2-anthracene)-N′-(4-phenylethyl-N′′-morpholine)-squaramide (**7**)

**Figure S22.** ^1^H NMR (400 MHz) spectrum of **7** in DMSO-*d_6_* at 298 K. Ethanol solvent peaks present in spectrum.

**Figure S23.** ^13^C NMR (101 MHz) spectrum of **7** in DMSO-*d_6_* at 348.15 K.

**Figure S24.** HR-MS (ESI^+^) spectrum of *N*-(2-anthracene)-*N′*-(4-phenylethyl-*N′′*-morpholine)-squaramide (**7**)

N-(2-anthracene)-N′-(4-phenylethyl-N′′,N′′-dimethylamine)-squaramide (**8**)

**Figure S25.** ^1^H NMR (400 MHz) spectrum of **8** in DMSO-*d_6_* at 298 K. Ethanol solvent peaks present in spectrum.

**Figure S26.** ^13^C NMR (101 MHz) spectrum of **8** in DMSO-*d_6_* at 298 K.

**Figure S27.** HR-MS (ESI^+^) spectrum of *N*-(2-anthracene)-*N′*-(4-phenylethyl-*N′′,N′′*-dimethylamine)-squaramide (**8**)

# 4. ^1^H NMR Titration Experiments

## 4.1. Sample Preparation

Proton NMR titrations were performed on a Bruker Ascend 400 NMR spectrometer. Stack plots were made using MestReNova Version 14.1.2. Tetrabutylammonium chloride was dried under high vacuum for 24 h to remove residual water. A stock solution of the host (2.0 – 3.0 mM) was prepared in DMSO-*d_6_* / 0.5% H_2_O. A portion of the host stock solution (600 µL) was transferred to an NMR sample tube. The same host stock solution was then used to prepare the standard guest titrant solutions containing approximately 0.1 M or 1 M TBACl, ensuring a constant concentration of the host for the duration of the titration experiment.

## 4.2. Titration Procedure

The proton NMR spectra of the host solution was collected at the start of the experiment. During the titration, small aliquots of the standard guest solution were added to the host solution in the NMR tube via Hamiltonian microlitre syringes. Before collecting each data point, an aliquot of the standard guest solution was added, and the sample was thoroughly mixed in the NMR tube. The system was allowed to equilibrate for up to 2 min inside the NMR probe before the spectra were collected. For each titration 17-18 data points were collected. The number of equiv. of guest present at the end of each titration is labelled in the stack plots.

## 4.3. Titration Data Fitting

Due to the highly hygroscopic nature of TBACl, even though the salts were dried under a high vacuum for 24 hrs prior to use in the experiments, water taken up by the salts during preparation of the samples can lead to inaccurate host:guest equivalents when calculated by mass. To confirm the host:guest equivalents in each titration, the TBACl NMR signals were integrated with respect to the known signals from the transporters. This was used to calculate the correct number of equivalents of TBACl present in each experiment, and this data was used in the fitting process. For each titration proton resonances were monitored for changes in their chemical shift, indicative of anion:receptor hydrogen bonding. Three resonances were followed, allowing several data sets to determine the association constant (K*_a_*). Global fitting considers all data sets simultaneously and improves the quality of non-linear curve fitting. The supramolecular.org web applet was employed to analyse the titration data, which demonstrated good fitting using a 1:1 host:guest binding model.^[14, 15]^

## 4.4 Titration Data with TBACl Guest

**Figure S28:** ^1^H NMR titration spectra as a stack plot for **1** (2.3 mM) + TBACl (0 – 120 equiv.) in DMSO-*d_6_* / 0.5% H_2_O at 298 K.

**Figure S29:** Fitted binding isotherm of **1** + TBACl in DMSO-*d_6_* / 0.5% H_2_O showing the change in chemical shift of NH protons fitted to a 1:1 binding model (left), K*_a_ =* 354 M^-1^. Residual plot showing the random error obtained from the binding isotherm fitting (right). Link to the Bindfit fitting: <http://app.supramolecular.org/bindfit/view/5622cf6b-c8e5-4956-8875-69f671ae350a>

**Figure S30:** ^1^H NMR titration spectra as a stack plot for **2** (2.7 mM) + TBACl (0 – 161 equiv.) in DMSO-*d_6_* / 0.5% H_2_O at 298 K.

**Figure S31:** Fitted binding isotherm of **2** + TBACl in DMSO-*d_6_* / 0.5% H_2_O showing the change in chemical shift of NH protons fitted to a 1:1 binding model (left), K*_a_ =* 468 M^-1^. Residual plot showing the random error obtained from the binding isotherm fitting (right). Link to the Bindfit fitting: <http://app.supramolecular.org/bindfit/view/cade3fc6-0acf-41b9-95cd-c9bc861384d3>

**Figure S32:** ^1^H NMR titration spectra as a stack plot for **3** (3.0 mM) + TBACl (0 – 104 equiv.) in DMSO-*d_6_* / 0.5% H_2_O at 298 K.

**Figure S33:** Fitted binding isotherm of **3** + TBACl in DMSO-*d_6_* / 0.5% H_2_O showing the change in chemical shift of NH protons fitted to a 1:1 binding model (left), K*_a_ =* 378 M^-1^. Residual plot showing the random error obtained from the binding isotherm fitting (right). Link to the Bindfit fitting: <http://app.supramolecular.org/bindfit/view/b72a7fab-45da-4d7e-8dd9-700e7f8c0687>

**Figure S34:** ^1^H NMR titration spectra as a stack plot for **4** (2.3 mM) + TBACl (0 – 113 equiv.) in DMSO-*d_6_* / 0.5% H_2_O at 298 K.

**Figure S35:** Fitted binding isotherm of **4** + TBACl in DMSO-*d_6_* / 0.5% H_2_O showing the change in chemical shift of NH protons fitted to a 1:1 binding model (left), K*_a_ =* 305 M^-1^. Residual plot showing the random error obtained from the binding isotherm fitting (right). Link to the Bindfit fitting: <http://app.supramolecular.org/bindfit/view/bf54b198-3a8a-4cbf-b2fd-3bdedd88fa11>

**Figure S36:** ^1^H NMR titration spectra as a stack plot for **5** (2.6 mM) + TBACl (0 – 100 equiv.) in DMSO-*d_6_* / 0.5% H_2_O at 298 K.

**Figure S37:** Fitted binding isotherm of **5** + TBACl in DMSO-*d_6_* / 0.5% H_2_O showing the change in chemical shift of NH protons fitted to a 1:1 binding model (left), K*_a_ =* 294 M^-1^. Residual plot showing the random error obtained from the binding isotherm fitting (right). Link to the Bindfit fitting: <http://app.supramolecular.org/bindfit/view/2bad39f5-0e23-4fbf-b140-f730412df76a>

**Figure S38:** ^1^H NMR titration spectra as a stack plot for **6** (2.1 mM) + TBACl (0 – 123 equiv.) in DMSO-*d_6_* / 0.5% H_2_O at 298 K.

**Figure S39:** Fitted binding isotherm of **6** + TBACl in DMSO-*d_6_* / 0.5% H_2_O showing the change in chemical shift of NH protons fitted to a 1:1 binding model (left), K*_a_ =* 546 M^-1^. Residual plot showing the random error obtained from the binding isotherm fitting (right). Link to the Bindfit fitting: <http://app.supramolecular.org/bindfit/view/d46d723a-300b-4982-becb-171490bc8d23>

**Figure S40:** ^1^H NMR titration spectra as a stack plot for **7** (2.5 mM) + TBACl (0 – 125 equiv.) in DMSO-*d_6_* / 0.5% H_2_O at 298 K.

**Figure S41:** Fitted binding isotherm of **7** + TBACl in DMSO-*d_6_* / 0.5% H_2_O showing the change in chemical shift of NH protons fitted to a 1:1 binding model (left), K*_a_ =* 137 M^-1^. Residual plot showing the random error obtained from the binding isotherm fitting (right). Link to the Bindfit fitting: <http://app.supramolecular.org/bindfit/view/b714a8c4-82fa-4c9c-a2ce-8b2856cd4608>

**Figure S42:** ^1^H NMR titration spectra as a stack plot for **8** (2.8 mM) + TBACl (0 – 144 equiv.) in DMSO-*d_6_* / 0.5% H_2_O at 298 K.

**Figure S43:** Fitted binding isotherm of **8** + TBACl in DMSO-*d_6_* / 0.5% H_2_O showing the change in chemical shift of NH protons fitted to a 1:1 binding model (left), K*_a_ =* 150 M^-1^. Residual plot showing the random error obtained from the binding isotherm fitting (right). Link to the Bindfit fitting: <http://app.supramolecular.org/bindfit/view/9a9f83e3-655d-4299-bf16-9344a6d8a501>

**Figure S44:** ^1^H NMR titration spectra as a stack plot for **SA2** (2.7 mM) + TBACl (0 – 100 equiv.) in DMSO-*d_6_* / 0.5% H_2_O at 298 K.

**Figure S45:** Fitted binding isotherm of **SA2** + TBACl in DMSO-*d_6_* / 0.5% H_2_O showing the change in chemical shift of NH protons fitted to a 1:1 binding model (left), K*_a_ =* 187 M^-1^. Residual plot showing the random error obtained from the binding isotherm fitting (right). Link to the Bindfit fitting: <http://app.supramolecular.org/bindfit/view/b4050fd3-31cf-41a3-8a24-11b84d26600f>

**Figure S46:** ^1^H NMR titration spectra as a stack plot for **SA3** (2.6 mM) + TBACl (0 – 95 equiv.) in DMSO-*d_6_* / 0.5% H_2_O at 298 K.

**Figure S47:** Fitted binding isotherm of **SA3** + TBACl in DMSO-*d_6_* / 0.5% H_2_O showing the change in chemical shift of NH protons fitted to a 1:1 binding model (left), K*_a_ =* 116 M^-1^. Residual plot showing the random error obtained from the binding isotherm fitting (right). Link to the Bindfit fitting: <https://app.supramolecular.org/bindfit/view/816b99c7-0b1d-4212-8699-a5f10fe46aa3>

# 5. HPTS Assay

## 5.1. Vesicle Preparation

POPC (1-palmitoyl-2-oleoyl-sn-glycero-3-phosphocholine), supplied by Corden Pharma Switzerland LLC, was stored at –20°C as a solution in chloroform (1g POPC in 35 mL chloroform). Triton X-100 was used as detergent and was supplied by Sigma-Aldrich.

Unilamellar vesicles were prepared following a procedure outlined previously by the Gale group.^[18]^ A ca. 38 mM chloroform solution of POPC (2 mL) was transferred to a pre-weighed round-bottom flask, and the solvent was removed using a rotary evaporator. The temperature was maintained at 35 ℃ and the pressure was lowered slowly to ensure the formation of a smooth lipid film. The film was then dried *in vacuo* for a minimum of 12 h, and the mass of lipid was recorded. The lipids were rehydrated by vortexing with 2 mL of the internal solution (100 mM NaCl, 10 mM HEPES, 1 mM HPTS, adjusted to pH 7.0) until all lipids were removed from the sides of the flask and were suspended in solution. The suspension was subjected to 9 freeze-thaw cycles by freezing in a liquid nitrogen bath and thawing in lukewarm water. Subsequently, the vesicles were left to rest at room temperature for 30 min. The lipids were extruded through a 200 nm polycarbonate membrane 25 times using an extruder set (Avanti Polar Lipids Inc) to form monodisperse vesicles. Only 1 mL of solution was extruded at a time before being collected.

Finally, any residual unencapsulated salt from the internal solution was removed via size-exclusion using a B19 column packed with hydrated G-25 Sephadex®, which had been pre-saturated with the external solution (100 mM NaCl, 10 mM HEPES, adjusted to pH 7.0). The lipid suspensions were diluted with the external solution to afford a stock solution (10 mL) of a known concentration.

## 5.2. HPTS Assay Hill Analyses

For a given experiment, the prepared vesicles were diluted to a concentration of 0.1 mM on a 2.5 mL plastic cuvette. An aliquot of aqueous NaOH solution (25 µL, 0.5 M) was added at approximately *t* = ­–30 s to increase the pH of the external solution by approx. one pH unit to pH unit to 8.0 to create a pH gradient and drive transport through the vesicle membrane. Transport was initiated with the addition of the transporter as a DMSO solution (5 µL) and ended with the addition of detergent (Triton X-100, 10% v/v in water, 50 µL) was added at *t* = 210 s to lyse the vesicles, and a final fluorescence intensity reading was recorded at *t* = 300 s to signify 100% efflux.

The changes in the fluorescent activity of intravesicular HPTS were used to detect pH changes during the experiments and represent proton efflux. The acidic and basic forms of the HPTS probe were excited at λ_ex_ = 403 nm and λ_ex_ = 460 nm, respectively, and the fluorescence emission of both forms recorded at λ_em_ = 510 nm. The intensity ratio of the basic form to the acidic form was determined, and the fractional fluorescence intensity (*I_F_*) was calculated using the equation:

$$I_{F}= \frac{R_{t}-R_{0}}{R_{d}-R_{0}}$$

**Equation S1.** Used for the conversion of raw HPTS data into fractional fluorescence intensity.

Where *R_t_* is the ratiometric fluorescence value at a given time (t), *R_0_* is the ratiometric fluorescence value at *t* = 0 s, and *R_d_* is the fluorescence ratiometric value recorded at *t* = 360 s following the addition of detergent.

Dose-response experiments were performed at a minimum of six transporter concentrations plus a blank DMSO control run. The fractional fluorescence intensity (*I_F_*) was plotted as a function of transporter concentration (mol%, with respect to lipid concentration). The *I_F_* value at *t* = 200 s for each tested transporter concentration was fit to an adapted Hill Equation, using *Origin 2021b (Academic),* given as:

$$y=y_{0}+(y_{max}-y_{0})\frac{x^{n}}{k^{n}+x^{n}}$$

**Equation S2.** The modified Hill equation employed in the HPTS assay.

Where *y_0_* is the *I_F_* value at *t* = 200 s for the DMSO blank run, *y_max_* is the maximum *I_F_* value, *n* is the Hill coefficient, and k is a derived parameter. A derived equation was used to calculate the EC_50_ value, the transporter concentration required to facilitate 50% chloride efflux, given as:

$$\mathrm{EC}_{50}=\left( \frac{{-k}^{-n}(0.5-y_{1})}{0.5} \right)^{-\frac{1}{n}}$$

**Equation S3.** The adapted EC_50_ equation.

Where *k* and *n* are the derived parameters from the Hill equation, and y_1_ is the last ratiometric value.

The dose-response curves and Hill analyses for transporters **1**–**8** are shown.

**Figure S48.** Efflux plot and Hill analysis of H^+^/Cl^-^ symport (or Cl^-^/OH^-^ antiport) facilitated by **1** in the NaCl HPTS assay. Each data point is the average of two repeats, with the error bars showing the standard deviation. A run of DMSO was used as a control.

**Figure S49.** Efflux plot and Hill analysis of H^+^/Cl^-^ symport (or Cl^-^/OH^-^ antiport) facilitated by **2** in the NaCl HPTS assay. Each data point is the average of two repeats, with the error bars showing the standard deviation. A run of DMSO was used as a control.

**Figure S50.** Efflux plot and Hill analysis of H^+^/Cl^-^ symport (or Cl^-^/OH^-^ antiport) facilitated by **3** in the NaCl HPTS assay. Each data point is the average of two repeats, with the error bars showing the standard deviation. A run of DMSO was used as a control.

**Figure S51.** Efflux plot and Hill analysis of H^+^/Cl^-^ symport (or Cl^-^/OH^-^ antiport) facilitated by **4** in the NaCl HPTS assay. Each data point is the average of two repeats, with the error bars showing the standard deviation. A run of DMSO was used as a control.

**Figure S52.** Efflux plot and Hill analysis of H^+^/Cl^-^ symport (or Cl^-^/OH^-^ antiport) facilitated by **5** in the NaCl HPTS assay. Each data point is the average of two repeats, with the error bars showing the standard deviation. A run of DMSO was used as a control.

**Figure S53.** Efflux plot and Hill analysis of H^+^/Cl^-^ symport (or Cl^-^/OH^-^ antiport) facilitated by **6** in the NaCl HPTS assay. Each data point is the average of two repeats, with the error bars showing the standard deviation. A run of DMSO was used as a control.

**Figure S54.** Efflux plot of H^+^/Cl^-^ symport (or Cl^-^/OH^-^ antiport) facilitated by **7** in the NaCl HPTS assay. Each data point is the average of two repeats, with the error bars showing the standard deviation. A run of DMSO was used as a control.

**Figure S55.** Efflux plot of H^+^/Cl^-^ symport (or Cl^-^/OH^-^ antiport) facilitated by **8** in the NaCl HPTS assay. Each data point is the average of two repeats, with the error bars showing the standard deviation. A run of DMSO was used as a control.

## 5.3. HPTS Assay BSA Mechanistic Studies

Untreated vesicle experiments were conducted by diluting the prepared vesicles to a concentration of 0.1 mM in a 2.5 mL plastic cuvette. An aliquot of aqueous NaOH solution (25 µL, 0.5 M) was added at approximately *t* = ­–30 s before the experiment was initiated with the addition of the transporter DMSO solution (5 µL). The concentration of the transporter was determined by the EC_50_ calculated using the HPTS NaCl assay. Detergent (Triton X-100 (10% v/v in water), 50 µL) was added at *t* = 210 s to lyse the vesicles, and a final fluorescence intensity reading was recorded at *t* = 300 s to signify 100% efflux. For experiments with the protonophore CCCP, the CCCP (5 µL, 0.5 mM) was added at approximately *t* = ­–30 s, resulting in 1 mol% CCCP.

Fatty acid-free vesicles were produced by stirring a portion of the vesicle-suspended solution for 30 min in the presence of 1 mol% bovine serum albumin (BSA), which sequestered the membrane-embedded fatty acids. The experimental procedure continued with the description outlined above for the untreated vesicles. Finally, a known quantity of fatty acids was added to the BSA-treated vesicles as an oleic acid solution (10 mol%) at *t* = ­–60 s.

# 6. pKa Data

| Compound | p*K*_a_ of targeting group N |
| --- | --- |
| 1 | 7.1 |
| 2 | 8.8 |
| 4 | 7.1 |
| 5 | 8.9 |
| 7 | 7.1 |
| 8 | 9.1 |

**Table S1.** p*K*_a_ of targeting group N for all targeted compounds calculated using MolGpKa.^[21, 22]^

# 7. Photophysical Studies

**Figure S56.** Excitation-emission spectra for transporters **6**–**8** in DMSO (1 % H_2_O), 10 µM.

# 8. Cell Culture

## 8.1. Cell Culture Conditions

Human MDA-MB-231 breast cancer cells (ATCC, RRID: CVCL_0062) and human BEAS-2B lung epithelial cells (ATCC, RRID: CVCL_0168) were cultured in Dulbecco’s Modified Eagle Medium containing 1% (v/v) penicillin/streptomycin (Merck) and 10% (v/v) fetal bovine serum (Thermo Fisher Scientific). Cells were cultured at 37 °C in a humidified atmosphere of 5% CO_2_ and harvested at 80% confluency using trypsin/EDTA after washing with Dulbecco’s phosphate-buffered saline (dPBS, Merck). Transporters were administered to cells using a DMSO vehicle (final concentration 0.5% v/v) and were compared against cells treated with DMSO alone (vehicle only control).

## 8.2. MTS Cell Viability Assay

Cell viability following 72-hour treatment with the transporters was assayed by measuring the conversion of MTS (3-(4,5-dimethythiazol-2-yl)-5-(3-carboxymethoxyphenyl)-2-(4-sulfonyl)-2H-tetrazolium) to formazan by dehydrogenase enzymes in metabolically active cells. The assay was carried out in both MDA-MB-231 and BEAS-2B cells.

In a 96-well plate, cells were seeded in triplicate in complete media at a seeding density of 5 x 10^3^ cells per well for MDA-MB-231, and 2 x 10^3^ cells per well for BEAS-2B cells. The cells were incubated for 24 h. Then media was removed and replaced with DMEM containing various drug concentrations and incubated for a further 72 h. Cells were then incubated with CellTiter MTS 96 Aqueous MTS Reagent Powder (Promega) and phenazine ethosulfate (Sigma Aldrich) under dark conditions for approximately 3 h. The absorbance of each well at 490 nm was measured using a Tecan Spark plate reader to evaluate cell viability (CellTiter 96 Aqueous Non-Radioactive Cell Proliferation Assay, Promega). Three independent experiments (*N* = 3) were performed for each compound, resulting in nine readings per concentration.

Dose-response curves were constructed using log(inhibitor) vs response, variable slope (4 parameters) nonlinear regressions on GraphPad Prism 8. Absolute IC_50_ concentrations were interpolated from these normalised curves (data normalised to DMSO vehicle control) with the top constrained to 100%. Equation: Y=Bottom + (Top-Bottom)/(1+10^((LogIC50-X)*HillSlope)).

**Figure S57.** Representative dose-response curves showing the antiproliferative effects of compounds **1**-**8** in MDA-MB-231 cells in a 72 h MTS cell viability assay.

**Figure S58.** Representative dose-response curves showing the antiproliferative effects of compounds **1**-**8** in BEAS-2B cells in a 72 h MTS cell viability assay.

## 8.3. Apoptosis and Necrosis Assay

The ability of the test compounds to induce apoptosis or necrosis was measured using the RealTime-Glo^TM^ Annexin V Apoptosis and Necrosis Assay (Promega), a plate-based kinetic assay that uses a luminescent annexin V probe to monitor phosphatidylserine exposure and a cell-impermeant profluorescent DNA dye to monitor loss of membrane integrity. The assay was performed in white solid bottom 96-well plates at 37°C using a temperature-controlled Tecan Spark multimode plate reader, using CO_2_-independent media formulations according to the manufacturer’s protocol. Before use, the inner surface of the plate lid was treated with a sterile 20% ethanol solution containing 0.05% Triton X-100 and dried fully to prevent condensation interference during the experiment.^[33]^

MDA-MB-231 cells were suspended in HEPES-buffered (25 mM, pH 7.2) low glucose Dulbecco’s Modified Eagle Medium (without bicarbonate) containing 10% (v/v) fetal bovine serum and 1% (v/v) penicillin/streptomycin (Merck) to a concentration of 200,000 cells/mL, then seeded in triplicate in white solid bottom 96-well plates (10,000 cells/well, 50 μL) and incubated for 4-5 hours. The test compounds were prepared as 0.5% DMSO solutions at 4X their desired treatment concentration and the 2X detection reagent was prepared immediately before treatment. Each well was treated with 50 μL of the test compound solution (or DMSO vehicle control) followed by 100 μL of the detection reagent. After mixing the well contents using a plate shaker (300 rpm, 30 seconds), the lidded plate was transferred into the pre-warmed pate reader to commence the experiment. An initial luminescence and fluorescence (485 nm_ex_ / 525 nm_em_) reading was recorded, followed by measurements taken every hour for the duration of the experiment. Three independent experiments (*N* = 3) were performed for each compound, with fluorescence and luminescence measurements reported as mean ± SD from triplicate wells.

**Figure S59.** Apoptosis and necrosis detection using the Promega RealTime-Glo^TM^ Annexin V Apoptosis and Necrosis Assay. Luminescence and fluorescence signals were measured over time to monitor early apoptotic (phosphatidylserine exposure) and late necrotic (membrane compromise) events, respectively.

# 9. Confocal Microscopy

## 9.1. Acridine Orange Staining

MDA-MB-231 cells were seeded at a density of 1 x 10^5^ into PhenoPlate^TM^ 96-well microplates (Revvity) and allowed to adhere for 24 h. The media was removed and replaced with 100 µL DMEM solutions containing 5 µM or 10 µM of the transporters with ≤ 0.5% DMSO. The cells were then incubated for 3 h. Acridine orange (5 μg/mL) was added and the cells were incubated for a further 30 mins. The cells were subsequently washed with warm PBS with 10% FBS (3 x 100 µL) and then imaged in 100 µL FluoroBrite DMEM (Thermo Fisher Scientific).

## 9.2. Colocalisation Imaging

MDA-MB-231 cells were seeded at a density of 1 x 10^5^ into PhenoPlate^TM^ 96-well microplates (Revvity) and allowed to adhere for 24 h. The media was removed and replaced with 100 µL DMEM solutions containing 5.5 µM or 3 µM of the transporters with ≤ 0.5% DMSO. The cells were then incubated for 3 h. Lysotracker Deep Red (50 nM) was added and the cells were incubated for a further 30 mins. The cells were subsequently washed with warm PBS (3 x 100 µL) and then imaged in 100 µL FluoroBrite DMEM (Thermo Fisher Scientific).

## 9.3. Leica Stellaris 8 Confocal Microscope

Images were obtained at 37 °C in 5 % CO_2_ atmosphere on a Leica Stellaris 8 inverted confocal laser scanning microscope, equipped with a HC PL APO CS2 40x water immersion objective.

For acridine orange staining a white light laser set to 498 nm was used. Emission was collected at 503 – 626 nm and 626 – 830 nm.

For colocalisation imaging, a 405 nm diode laser and white light laser set to 653 nm were used. Emission was collected at 430 – 600 nm and 660 – 830 nm.

## 9.4. Image Data Analysis

Images were collected using LAS-X (Leica) software and the imaging data was analysed and processed using ImageJ/FIJI (version 1.54p).^[34]^ Pearson’s correlation coefficients were calculated using the JACoP BIOP and Colocalization Finder plugins and is reported as the mean from at least three images.

**Figure S60*.*** Representative images of live MDA-MB-231 breast cancer cells dosed with 5.5 uM of compound **6** for 3 hrs, followed by LysoTracker Deep Red (LTDR) (50 nM) for 30 mins. A. Cyan channel (Transporter): λ_ex_ = 405 nm, λ_em_ = 430 – 600 nm. B. Magenta channel (LTDR): λ_ex_ = 653 nm, λ_em_ = 660 – 830 nm. C. Composite image. D. Scatterplot from the colocalisation analysis. All scale bars represent 20 μm.

**Figure S61.** Representative images of live MDA-MB-231 breast cancer cells dosed with 5.5 uM of compound **7** for 3 hrs, followed by LysoTracker Deep Red (LTDR) (50 nM) for 30 mins. A. Cyan channel (Transporter): λ_ex_ = 405 nm, λ_em_ = 430 – 600 nm. B. Magenta channel (LTDR): λ_ex_ = 653 nm, λ_em_ = 660 – 830 nm. C. Composite image. D. Scatterplot from the colocalisation analysis. All scale bars represent 20 μm.

**Figure S62.** Representative images of live MDA-MB-231 breast cancer cells dosed with 5.5 uM of compound **8** for 3 hrs, followed by LysoTracker Deep Red (LTDR) (50 nM) for 30 mins. A. Cyan channel (Transporter): λ_ex_ = 405 nm, λ_em_ = 430 – 600 nm. B. Magenta channel (LTDR): λ_ex_ = 653 nm, λ_em_ = 660 – 830 nm. C. Composite image. D. Scatterplot from the colocalisation analysis. All scale bars represent 20 μm.
